# Trends of Human-Robot Collaboration in Industry Contexts: Handover, Learning, and Metrics

**DOI:** 10.3390/s21124113

**Published:** 2021-06-15

**Authors:** Afonso Castro, Filipe Silva, Vitor Santos

**Affiliations:** 1 Department of Mechanical Engineering (DEM), Institute of Electronics and Informatics Engineering of Aveiro (IEETA), University of Aveiro, 3810-193 Aveiro, Portugal; vitor@ua.pt; 2 Department of Electronics, Telecommunications and Informatics (DETI), Institute of Electronics and Informatics Engineering of Aveiro (IEETA), University of Aveiro, 3810-193 Aveiro, Portugal; fmsilva@ua.pt

**Keywords:** Human-Robot Collaboration, interfaces of communication, physicality, object handover, robot cognition

## Abstract

Repetitive industrial tasks can be easily performed by traditional robotic systems. However, many other works require cognitive knowledge that only humans can provide. Human-Robot Collaboration (HRC) emerges as an ideal concept of co-working between a human operator and a robot, representing one of the most significant subjects for human-life improvement.The ultimate goal is to achieve physical interaction, where handing over an object plays a crucial role for an effective task accomplishment. Considerable research work had been developed in this particular field in recent years, where several solutions were already proposed. Nonetheless, some particular issues regarding Human-Robot Collaboration still hold an open path to truly important research improvements. This paper provides a literature overview, defining the HRC concept, enumerating the distinct human-robot communication channels, and discussing the physical interaction that this collaboration entails. Moreover, future challenges for a natural and intuitive collaboration are exposed: the machine must behave like a human especially in the pre-grasping/grasping phases and the handover procedure should be fluent and bidirectional, for an articulated function development. These are the focus of the near future investigation aiming to shed light on the complex combination of predictive and reactive control mechanisms promoting coordination and understanding. Following recent progress in artificial intelligence, learning exploration stand as the key element to allow the generation of coordinated actions and their shaping by experience.

## 1. Introduction

The industry world uses many automation systems to achieve massive production by the repeatability that it allows. However, traditional robotic systems are not suitable for every task that each production requires. There are some tasks that involves uncertainties and demand cognitive knowledge, that only humans can provide, to successfully complete them. Contrarily, humans do not have the same speed, stamina and physical strength as robots do. This duality leads to an idealization of a co-working between humans and robots in a way that both of the contributors could make use of their own values and not be limited by their own conditions. This combination of working is addressed as Human-Robot Collaboration (HRC).

In [1], numerous applications of robots within human lives are presented, involving topics such as elderly, bio-feedback system, schools and learning, medicine, entertainment, space exploration and military. Several studies and research works were recently developed contributing to the progress of this research field. The work of Ajoudani et al. [2] shows the remarkable growth of publications between the years 1996 and 2015 regarding the topic of Human-Robot Collaboration. As expected, each robotic researcher provides a subjective consideration about HRC. For example, Hentout et al. [3] sustain that a collaborative robot should improve the quality of task execution, ensure safety for the human user and be ergonomic for this same human co-worker. According to Zaatari et al. [4], HRC translates a balance between automation and flexibility, aiming to respond to the demands of the market in terms of product customization, variability, and cycle time. These different perspectives fall on the distinct levels that the HRC domain enclose.

There are several research challenges that need to be addressed in the study of collaborative processes that are at the intersection of robotics, engineering, Human-Robot Interaction, and computer science. Collaborative robots need to be endowed with a set of abilities that enable them to act in close contact with humans, such as sensing, reasoning, and learning. In turn, the human must be placed at the centre of a careful design where safety aspects and intuitive physical interaction need to be addressed as well. Industrial applications impose additional concerns since the robots need to be easily programmed by non-expert users, while operating in poorly structured, dynamic environments. The development of intuitive and natural communication interfaces requiring little training and capable of maintaining low levels of fatigue is a priority, including those associated with direct physical interaction.

**Figure 1:**
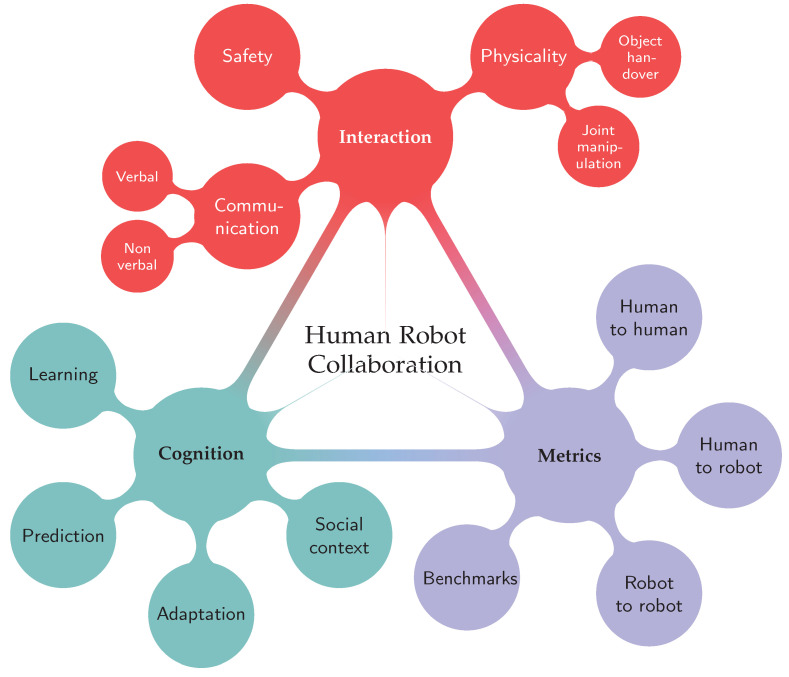
Mind map of the Human-Robot Collaboration paradigm.

Considering the ultimate goal of a fluent, human-like and flawless collaboration, we realize the existence of a wide range of topics that need to be completely covered up, such as safety, interaction, physicality, cognition, adaptation, metrics, among many others. Figure 1 provides a hierarchical representation of some of the fundamental topics associated with HRC, giving a special emphasis to the following research trends: better interaction, cognitive integration, and effective metrics. Despite the continuous advances, new techniques for improved functionalities need to be developed with safe physical interaction in mind and validated in industrial scenarios.

### 1.1. Analysis of Past Reviews

In the last few years, several review articles on HRC have appeared that provide a summary of previous research and a comprehensive view of this wide research field. This section provides a short analysis of past review articles in an attempt to clarify the differences in the structure and type of selected information, as well as in the depth to which the topics are covered. Ajoudani et al. [2] reviewed the state-of-the-art on technologies and methodologies enabling achieving advanced human-robot collaborative systems. That paper describes in a very complete way the bi-directional human-robot interfaces and interaction modalities, as well as adaptive control methodologies to link perception to robot behaviour, namely for setting up a successful physical HRC (pHRC) framework. Additionally, the authors discuss the challenges of control performance in terms of stability and transparency, including useful lessons from the field of human motor control. Potential applications and relevant use cases are also presented, jointly with benchmarks regarding acceptance aspects.

Villani et al. [5] provide an extensive review of HRC for industrial environments, focusing on problems related to physical and cognitive interaction. Safety standards are presented from the perspective of requirements and design guidelines useful for development of new systems. The main body of the paper is on state-of-the-art approaches for developing intuitive user interfaces, including interaction modes and robot programming in manufacturing practice. The main industrial applications where collaborative robots may improve task efficiency are also discussed. In [4], El Zaatari et al. address the programming requirements for effective collaboration in industrial settings. The review is organized around three important aspects: communication, optimization, and learning. Communication between humans and robots is categorised in body language and speech, user interfaces and haptics. The optimization approach is centred on of different aspects related to the comfort of the human operator and task efficiency. Research studies exploring learning to allow generation of coordinated actions are also discussed, with emphasis on learning from demonstration and reinforcement learning techniques.

Other review articles are organized in a chronological perspective that helps to analyse the trends observed in recent years. Matheson et al. [6] conducted a literature review of collaborative robotics in manufacturing or assembly applications, covering the period from 2009 to 2018. The selected literature comprises a total of 35 case studies where practical experiments with real hardware are conducted. The analysis of the reviewed literature summarizes each individual study, providing information such as the robot used, the control system, the collaboration methodology, the industrial application, as well as the objective in HRC research and the key findings. Hentout et al. [3] provide a broad coverage of the state-of-the-art on industrial collaborative robotics by reviewing more than 300 papers published between 2008 and 2017. For that purpose, authors classify the content of these papers into seven research categories, as follows: “hardware and software design of cobotic systems”, “safety in industrial cobotics”, “cognitive human-robot interactions”, “robot programming approaches”, “human-robot task allocation”, “virtual and augmented reality”, “study of physical interactions between humans” and “fault tolerance”. In addition to this classification, each selected article is grouped in several sub-categories, while the proposed approach, and the main results are summarized.

In a recent review, Kumar et al. [7] give an overview of Human-Robot Collaboration in industrial environments centred around three challenges facing the development of collaborative systems: human safety, trust in automation (i.e., user’s expectations and acceptance) and productivity impact. Beyond the focus on human safety, this survey proposes a categorization of HRC based on three aspects of the system: awareness (perception and sensors), intelligence (robot action and behaviour), and compliance (control interfaces and communication). Another very recent review covering the same topic can be found in [8]. In this paper, Ogenyi et al. describe robotic systems with cutting edge technologies in terms of sensors and actuators suitable for pHRC. The survey reveals relevant components for effective pHRC to be accomplished, including the adoption of sophisticated learning techniques and control algorithms. Collaborative strategies are discussed from the viewpoint of attention formulation, coordination mechanisms and task planning. Learning methods useful for pHRC are also briefly discussed, including human action recognition, human motion prediction, learning control policies, and skill transfer from humans to robots.

### 1.2. Purpose and Contribution

Robotics is currently undergoing a paradigm shift guided by technological advances such as the human-centred design, enhanced sensing capabilities and increased computing power. This paper aims to highlight meaningful research trends that can help leverage the potential of HRC in the automation domain. As a result, a particular focus lies on three key principles that should be considered together as a way of promoting future developments—better interactions, cognitive integration, and effective metrics.

First, physical interactions are a likely occurrence in industrial collaborative work, and object handovers, in particular, will play an important role. Closed interactions with humans will be a key ability of the next generation of collaborative robots. Second, the integration of cognition would enable the robot to interpret the current situation, to remember experience, to predict future conditions and, as result, to influence its own action in real time. These abilities are important to make the system act proactively, while expressing an anticipatory and adaptive behaviour. Finally, metrics and benchmarks are also essential to establish the state of the art and, in this way, future progress. Thus, we seek to highlight the unique challenges of assessing performance of collaborative systems and the ongoing efforts.

In this context, the contribution of this paper is two-fold. On the one hand, we attempt to strengthen the links between research topics that are often addressed separately: handovers, learning and metrics. On the other hand, we provide an overview of publications that point to trends, research directions and next priorities and, to that extend, promise to reshape the field of HRC.

### 1.3. Paper Organization

The purpose of this work is to understand the state of the art of HRC research and to identify the scientific gaps that still hold the integration of robots in humans co-working teams. In line with this, the paper starts, in Section 2, with a literature overview about HRC in industrial contexts. This section defines the basic concepts, enumerates the most common interfaces of communication between humans and robots, and describes the physical interaction that Human-Robot Collaboration entails. Section 3 focuses on current advances and trends associated with handovers, learning and metrics. Seeking to study the critical moment of physical contact, an analysis about human-robot object handover developments is presented. The object transfer between the operator and the machine expresses the ultimate challenge to achieve an efficient and useful co-working. Recent works have been addressing learning techniques integration as the solution to reach the aimed fluency and coordination: the progress of combining artificial intelligence with collaborative robots are also explored in this work. Moreover, multiple metrics for Human-Robot Collaboration are examined. For a proper evaluation of each result, these metrics should be general, in a way that could also be applied to human-human collaboration, and agnostic to any procedure. Section 4 discusses the future challenges that should be tackled to achieve the intended collaboration between humans and robots, trying to narrow the open path for advances in this wide domain and, more specifically, in human-robot object handover. Finally, the main conclusions of this work are summarized in Section 5.

## 2. Human-Robot Collaboration Concepts

Before any discussion about the recent work and research, as well as the future opportunities that the collaboration between humans and robots could bring to humanity, several concepts that might be not so well clarified should be elucidated. It is known that, among all the areas where robots can provide useful and powerful help to humans, the industrial environment is the one where the quickest integration is expected, since human-robot co-work is already widely used in industrial tasks [1]. This expected significant integration of robots in humans life leads to a lot of interest and discussion among many robotic research laboratories which consequently, originated distinct, and often, misunderstanding concepts. For the purpose of this work, the definition and description of each robotic concept plays an essential role to allow a clear discussion about near future challenges and opportunities enclosed by HRC. This section provides an extensive review and interpretation of the HRC related issues, starting out from its own definition, enumerating several human-robot available communication channels and, lastly, deepening into the recent physical interaction interest between human and robots.

### 2.1. Interaction vs. Collaboration

The terms of *interaction* and *collaboration* between humans and robots are extensively used by numerous researchers in this particular topic of interest. Furthermore, Human-Robot Interaction (HRI) and Human-Robot Collaboration (HRC) are two easily recognized concepts regarding the human-robot co-work. These two concepts could be often confused with each other, or even be perceived as the exact same proposition. Since HRI and HRC symbolize relatively recent research fields, there is not yet a global and absolute definition of these two acronyms. However, the related literature starts to converge into the same interpretation, which is gathering all robotic researchers. For the best of our knowledge, it becomes fundamental to understand the differences between *interaction* and *collaboration* and, consequently, to correctly distinguish Human-Robot Interaction from Human-Robot Collaboration.

First, before mentioning any robotic participation, the *interaction* and *collaboration* words carry distinct meanings. Accordingly to the Cambridge Dictionary, *interaction* is defined as “an occasion when two or more people or things communicate with or react to each other” while *collaboration* is defined as “the situation of two or more people working together to create or achieve the same thing”. In the work of Grosz in 1996 [9], other dictionary definitions are provided for the interpretation of these two concepts: *interaction* is described as an action on someone or something else, while *collaboration* stands for the work “with” others: working jointly with someone or something. Grosz also focuses on the “jointly with” to distinguish *collaboration* from *interaction*. Several years later after the work of Grosz, Green et al. [10] also characterized *collaboration* as “working jointly with others or together especially in an intellectual endeavor”. As we can understand, this work perceives a similar meaning for *collaboration* as stated by Grosz [9]. A new interpretation perspective was introduced in [11], where the concept of *interaction* plays a more general role that includes *collaboration* within it. By presenting *interaction* as, once again, an “action on someone else”, this work refers to it as any kind of action that involves another human being or robot. Additionally, *collaboration* stands for “working with someone”, aiming at reaching a common goal. Taking these two considerations into account, it becomes easy to understand the way that [11] mentions *collaboration* as one particular case of *interaction*.

In an exclusively robotic field, ref. [12] defines *collaboration* as a robot feature to perform complex tasks with direct human interaction and coordination. According to [12], an interactive procedure can be perceived as a few nested behaviors that the robot must ensure: every collaboration implicates a coexistence that, in its place, implicates safety. As was already mentioned, ref. [11] encapsulates every collaboration within the concept of *interaction*; now, ref. [12] is relating *collaboration* to coexistence and safety.

In the work of Ajoudani et al. [2], a new definition of physical Human-Robot Collaboration (pHRC) is introduced: pHRC is the moment when human(s), robot(s) and the environment come to contact with each other and form a “tightly coupled dynamical system to accomplish a task”. In the same thought of the required machine capabilities to build a human-robot collaborative system, ref. [13] defines a collaborative robot as one that is able to understand its collaborator’s intentions and predict his/her actions, in order to adapt its behavior in accordance and provide assistance in a wide diversity of tasks. Furthermore, ref. [13] mentions different levels of robot autonomy since, for the authors, it should be capable of deciding when it can lead the task or instead follow the human. In Villani et al. [5], a distinction between Human-Robot Interaction (HRI) and Human-Robot Collaboration (HRC) is described, using two research works that were mentioned earlier: HRC requires a common goal that is sought by both robot and human working together ([11]); contrarily, in HRI the interaction between the human and the robot does not necessarily entail a common goal, thus falling in the definition of coexistence of [12].

From a different perspective, ref. [6] provides a clear definition of four interaction types: coexistence, synchronised cooperation and collaboration. The term of *coexistence* characterizes the moment when the human operator and the robot are in the same environment but do not interact with each other; *synchronised* describes the environment where the human and the robot work in the same space but at different times; *cooperation* is when the human and robot work on separate tasks, but in the same space at the same time and, lastly, *collaboration* is used when the human operator and the robot work together on the same task. Following the definitions of [6], when the robot is collaborating with the human, the action of the one has immediate consequences on the other. All this extended literature overview of the definitions about the Human-Robot Interaction and Human-Robot Collaboration concepts are summarized in Table 1.

**Figure 2:**
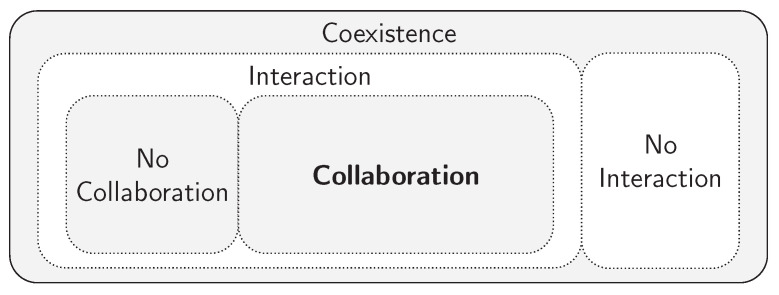
Relations between the different concepts about human-robot co-working.

With all these perspectives in mind, it is possible to understand the difference and deduce a clear interpretation about this particular topic. For the purpose of this work, and for future references, we will address the term of *interaction* as the communication between two entities. In other words, *interaction* describes the moment when someone gives any kind of information and someone else reacts accordingly. In a general point of view, *interaction* could be directly compared to a request-response service. On the other hand, *collaboration* describes the particular duties where human and robot help each other in the same task. At this moment of collaboration, no one in particular leads or follows: every collaborator could be leading or following the process, and its condition will probably change several times within the same task. Hence, any collaborative job implies numerous interactions. From another level, each interaction signifies a coexistence of the two intervening beings. Nonetheless, it is possible to have a human and a robot working in the same environment (coexistence) but without any interaction. In fact, these are the most used robotic systems by the industry world. The set of relations concerning these concepts is represented in Figure 2.

### 2.2. Interfaces of Communication

For a proper collaboration between a human operator and the robot, it is required that, at least, one of them communicates with the other. Accordingly to [14], if effective communication channels between humans and robots are established, then it becomes possible to release human workers from heavy tasks with assisted human/robot collaboration systems. Before enumerating the different communication channels, it is advisable that a definition of communication should be first clarified to understand the meaning and importance of interfaces of communication.

One definition can be found in [15], where communication stands as “a collection of mutual, common, or joint knowledge, beliefs, and suppositions”. For the authors of [15], every participant of a conversation changes his/her own mental state accordingly to their mutual current tasks and intentions understanding. In our perspective, any communication requires two active agents, and it is composed by the two moments of capturing data and interpreting the captured data. This means that it is crucial to have a normalized language or code (known by the two agents) that follows certain rules to ensure that a communication is established. The receptor should interpret the captured data in accordance to the known communicating code, to properly receive the information that the active agent provided. The easiest example where it is possible to understand this concept is the usual verbal human-human communication: the human ears are responsible to obtain the sounds emitted by the other human, and the knowledge of the spoken language will ensure the correct interpretation of the message. If the spoken language is not recognized by the receptor, even with a sensor capable of receiving the data, it becomes impossible to understand the message. Summing up, each interface of communication includes the received data and its classification or interpretation.

As defined previously, in order to complete each task successfully, a collaborative system always requires mutual interactions between the human and the robot. These interactions could be performed via several communication channels that are described in this subsection. Moreover, accordingly to [8], a robot can communicate contents, acquire knowledge about its surroundings, and give feedback to the environment or users, through these multiple communication interfaces. Some works have already categorized the several communication channels. In [10], three main types of communication between humans are indicated and detailed: audio, visual and environmental. However, as was previously stated, we will not consider the surroundings perception as a communication, since this kind of received information does not originate from another active agent with the intention to perform communication.

**Figure 3:**
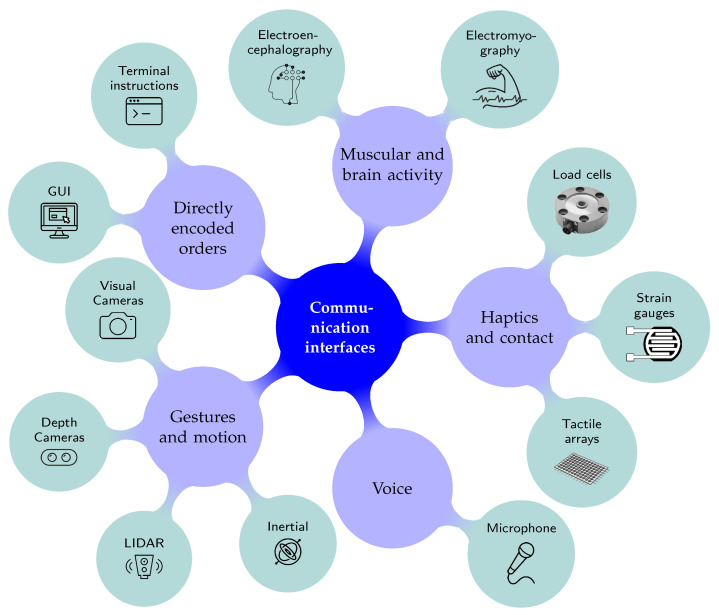
Overview of communication interfaces for HRC and some associate base sensors.

Currently, many different communication interfaces and methodologies can be applied to a Human-Robot Interaction system, allowing the communication between two agents. Figure 3 provides an overview of several communication interfaces: gesture recognition [14,16,17,18,19,20], human motion recognition [21,22], voice/speech recognition [15,16,23,24,25,26], haptics and contact sensors [8,23,27,28,29,30], EMG/EGG sensors [8,31,32,33,34] and GUI [35,36,37]. All these mentioned works use each type of interface for Human-Robot Interaction. Figure 3 also summarizes the common sensors that are used for each one of the communication channels. Since physical contact stands as the ultimate type of human-robot communication, especially for human-robot collaborative systems, physical interaction will be further discussed in the next subsections.

One of the most usual communication channel between humans and robots is presented in by Liu and Wang [14]; for these authors, hand gestures and body postures stand as effective communication channels in human-human collaboration. For this reason, their research dives into this particular interface of communication, enumerating three types of gestures: body gestures (full body actions or motions); hand and arm gestures (arm poses, hand gestures), and head and facial gestures (nodding or shaking head, winking lips). Additionally, ref. [14] proposes a workflow of the steps for communication interpretation by the robot. First, robot sensors have to capture the gesture raw data (*sensor data collection*); secondly, a gesture must be identified within all the data for each frame (*gesture identification*); thirdly, the identified gesture is tracked during the movement (*gesture tracking*); fourthly, according to pre-defined gestures type, the tracked gesture movement is classified (*gesture classification*); lastly, the robot translates this gesture recognition into robot commands (*gesture mapping*). All these steps seem very explicit an understandable, and can be, with small adjustments, extrapolated for all types of communication interfaces.

Another interface of communication is established by the combination of microphones and voice recognition algorithms. This type of communication is possibly the most similar to the usual communication between humans. However, an important issue regarding voice communication is recognized in [23]: in an industrial environment, the presence of background noise and chatter can make it problematic to perceive speech commands. From the same concern about the industrial adverse environmental conditions for a good performance of vision-based and/or voice-based interaction systems, ref. [16] presents an approach that relies on the speech and gesture recognition, dealing with information that can potentially be contradictory or complementary.

Apart from these two particular topics, a distinct channel of communication between humans and robots is also discussed in [8]: electromyography (EMG) and electroencephalography (EEG) sensors. When a muscle contracts, electrical signals are generated and measurable by an EMG sensor. EEG signals are the most used ones for brain-computer interfaces (BCIs).

In [4], in addition to all the mentioned communication channels, graphical user interfaces (GUI) are also included as a type of interaction between the human and the robot. The author states that an operator can control a collaborative robot through a communication interface that can be verbal (speech) or nonverbal. Nonverbal communication includes gestures, gaze, head pose, haptics and UIs. Similarly to the previous study, ref. [4] provides, in a much more summarized way, the general workflow that the collaborative robot must follow to communicate: signals are detected, recognised and mapped to executable actions for the robot. Notice that, by using a GUI, there is no need for sensors or for data recognition and classification algorithms, since the provided information is directly received by the robot, in a “language” that it knows since it was built.

A new type of interaction is also described in [8]: physical Human–Robot Collaboration (pHRC). In this type of collaboration, it becomes imperative for the robot to be able to observe its surroundings in order to take cognizance and continuous update of the current state. Based on that information, the robot could be endowed with the ability to estimate desired actions to be performed and the best possible way to perform them. In the example presented in [8], the human and the robot are performing a table-lifting task. For the successful completion of the task, the collaborative robot should identify the items in the environment (such as the table) and predict human future intention in order to act accordingly. Robotic skin tactile sensors (haptics) is also addressed in [8]. Robotic systems could be capable of identifying objects via multiple contact points thanks to the direct feedback that this technology provides. In the same study, it is possible to understand the technical issues associated to multiple conventional flexible sensors. For example, mechanical stress and aging can lead a reduction on the sensor’s sensitivity. The concept of *physical collaboration* is also explored in [12], where it is defined as “an explicit and intentional contact with exchange of forces between human and robot”. This exchange of forces establishes a communication channel since the robot can react accordingly or even predict human motion intentions just by measuring and estimating those forces. Moreover, the indicated work also mentions a contactless interaction that is based and guided through information exchange via direct communication, such as gestures and/or voice commands, or indirect communication by recognizing intentions through eye gaze, for example. Another study that address this communication interface is presented in [5], where it is possible to infer that, by the intentional establishment of a physical contact with exchange of forces between the two agents, or without contact (for example by the use of gesture or voice commands), a collaborative system capable of performing complex task alongside with the human can be achieved.

Another interesting issue to discuss in this topic of interfaces of communication is the level of autonomy of a robot. In fact, some collaborative robot systems give the possibility for the user (the human) to choose, by any kind of interaction (GUI, gesture, voice, etc.), when help from the robot is required or simply to stand still. Additionally, the human can also choose the level of collaboration: if the robot is allowed to predict human action and, thus, perform a collaboration task, or if the robot should just respond to specific request, such as a regular tool. Obviously, this difference between robot’s freedom has direct consequences in its motion planing and control.

### 2.3. Safety in HRC

One major concern regarding the application of human robot collaboration in industrial contexts is the fundamental problem of safety for both human operators and robots. Safety plays an essential role that should never be forgotten when studying human-robot co-working: every coexistence between a human and a machine entails a prior human safety. In fact, some standard ISO’s have been established (ISO 10218-1 and 10218-2) to ensure the operator safety when working with collaborative robots ([3]). These authors detail several safety measures when presenting the design of some cobots (collaborative robots), inferring that a robot must satisfy at least one of the four criteria presented and deeply described in [5], as follows: (i) *safety-rated monitored stop*—the cobot completely stops if an operator enters the workspace, (ii) *hand guiding*—the cobot is compliant and moved manually by the human, (iii) *speed and separation monitoring*—the closer a human operator becomes the slower the cobot moves, and/or (iv) *power and force limiting*—the cobot is programmed to operate only within tolerable levels of force and torque. These four levels of safety strategies, derived from ISO 10218-1 and 10218-2 standards are also explored in [4].

**Figure 4:**
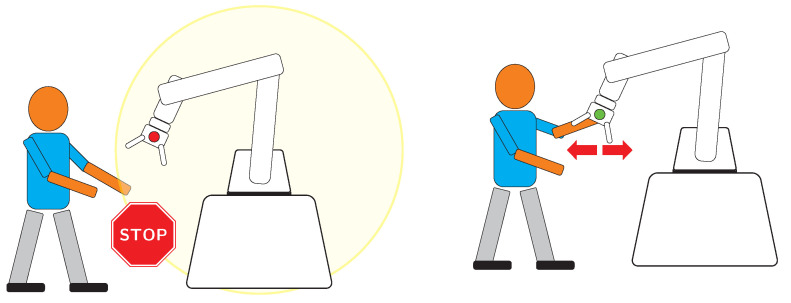
Illustration of the first levels of robot safety standards, as defined in norms ISO 10218-1 and 10218-2: the “Safety-rated monitored stop” on the left and the “Hand guiding” on the right.

**Figure 5:**
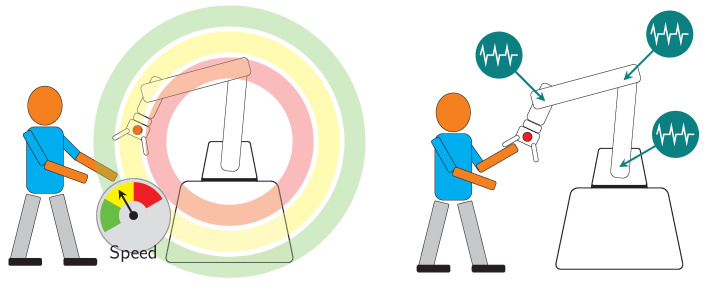
Illustration of the two more complex levels of robot safety standards, as defined in norms ISO 10218-1 and 10218-2: the “Speed and separation monitoring” on the left and “Power and force limiting” on the right.

The norms ISO 10218-1 and 10218-2 define several levels of safety strategies. The first two levels, with simpler definitions, level 1 and 2, as illustrated in Figure 4 are named “Safety-rated monitored stop” and “Hand guiding”. The other two levels, which encompass more complex requirements on the robot side, are level 3 and 4, named “Speed and separation monitoring” and “Power and force limiting” respectively, are illustrated in Figure 5.

Safety emerges as a still open research field, despite the numerous studies that already had tackled this particular issue. We refer the reader to the following reviews on the requirements and challenges of safety in HRC [3,5,7,38,39,40,41,42,43]. Some reported studies approach the safety problem by assuming the human operators and the robots share the same workspace but perform different tasks. In these cases, the robot should be able to track the motion of the human operator to estimate the distance between them and, thereafter, avoid any collision. For example, in order to avoid possible collisions, before any movement, a robot should plan its own trajectory and verify if it is all clear. Keeping a predefined distance between the robot and the human is a safety measurement that will, in all motions, interfere with the robot’s path planning. Different types of sensors and several strategies have been adopted to avoid potential collisions by jointly considering aspects of human monitoring and motion planning [44,45,46,47,48,49,50,51,52,53,54,55,56,57,58]. When it comes to physical human-robot interaction (pHRI), ref. [12] presents an approach to safe oriented robot control. The proposed application is based on an elaboration of physical collision detection and robot reaction by using a collision avoidance methodology. In certain safe coexistence conditions, the human has the possibility to activate or deactivate, using gestures, this safe physical collaboration approach.

### 2.4. Physical Interaction

A key concept regarding Human-Robot Collaboration is physicality. The vast majority of collaborative tasks entail a mutual touch in the same physical object, as both agents have a common goal to achieve. Many works and studies were performed in the scope of this concern, always in the attempt to reach the most efficient and intuitive Human-Robot Collaboration system.

Accordingly to [59], robotics has already a wide variety of tactile (touch) sensors. However, it is still hard to find an effective use of tactile sensors in robotics applications. One of the requirements for using tactile sensing in robotics is that all the robot body (skin) should be covered with sensors, just like the human skin. This distribution will endow the robot of decentralized and distributed control. This type of requirement creates a difficulty to turn tactile sensor modality into a reality. For [59], issues such as sensor placement, electronic/mechanical hardware, methods to access and acquire signals, automatic calibration techniques, and algorithms to process and interpret sensing data in real time must be tackled in order to reach to this efficient tactile sensible robot creation. In the literature studied by [59], the authors enumerate several tactile sensors capable of handling and measuring various contact parameters, such as: resistive, capacitive, optical, magnetic, ultrasonic, piezoelectric, electrorheological and magnetorheological sensors. In a more higher level, ref. [59] distinguishes different types of physical contact with objects: manipulation (perception for action), exploration (action for perception) and reaction/haptics (action-reaction).

A distinct concern in robotic physical interaction is mentioned in [60]: the object shape. Object shape plays a crucial role in robot grasping tasks. There exist multiple ways to obtain a object shape, but accurate models of novel objects shape are not so easy to achieve due to incomplete and noisy sensory measurements. Moreover, there are some type of object materials that are characterized for being deformable with frequent interaction. Bjorkman et al. [60] propose a probabilistic methodology for learning object models based on visual and tactile perception through physical interaction with an object. Through touch (physical interaction) at parts that are uncertain in terms of shape, the robot explores unknown objects. For the same concern, ref. [61] relies on dealing with uncertainties about physical properties of objects, such as the object weight and the friction at the points of contact. The proposed grasp adaption in [61] is based on an object-level impedance controller and a grasp stability estimator. The results of this approach show that the grasping performance on novel objects with distinct physical properties was improved. It is important to notice that not all issues regarding object grasping were tackled in [61]: some properties such as the coefficient of friction, object weight and center of mass must also be considering when, for example, lifting an object. For this reason, not only impedance controllers but also force controllers and position controllers must be implemented. Even so, uncertainty from finger dynamics and object geometry makes it very difficult to precisely control the grasping forces on the fingertips in real scenarios.

In a medical care context, ref. [62] reviews the literature of tactile sensation for upper-extremity prostheses, discussing several tactile sensing techniques. For [62], an ideal hand prosthesis should deliver tactile feedback in a natural manner and be reliable when decoding of the user’s intentions. That study also presents the characteristics of various tactile sensors such as strain gauges, piezoresistors, capacitive, piezoelectric and optical sensors. The authors of [62] conclude that all the tactile feedback techniques and devices have still limited success in clinical use. It is defended that more effort on compatibility with human skin artificial sensors should be carried out. For a proper estimation of object properties, interactive perception approaches must be performed. This kind of active perception approaches are defined, in [24], by a combination of physical interaction and traditional perception methods. This combination enables learning of object properties or even to manipulate unknown objects, being useful to numerous applications.

**Figure 6:**
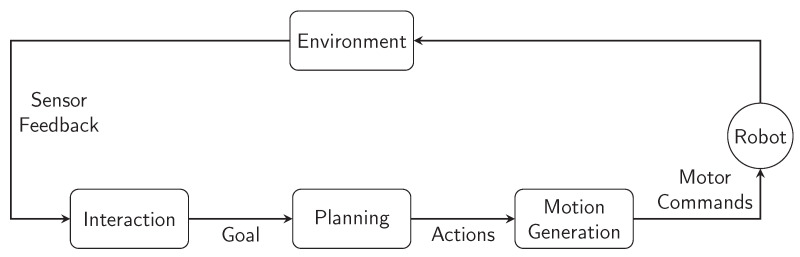
Scheme of the different layers of a generic human-robot interaction system.

Production, warehouse logistics, and construction are just some of the many domains where handling large objects plays a crucial role. Robotic systems specifically created for this purpose are mainly designed to operate autonomously without the need for human intervention ([63]). For an efficient Human-Robot Collaboration, these systems must be capable of understanding human goals and intentions, and quickly adapt themselves in accordance to the behavior of their coworkers. Furthermore, the robot’s judgement about who should lead the process should be inherent: the system must know when it can act on its own and when it should wait for human’s input information. Taking all this in consideration, ref. [63] proposes a procedure to cooperatively manipulating large objects by a human and a robot. By creating a physical interaction system, it becomes possible to handle or transport large objects, with distinct shapes, in collaboration with a human. In [63], a haptic feedback is used to infer which way the human will rotate the object and whether the user is ready to support the object. The robot plans its actions sequence in accordance to the rotation that is being proposed by the human. The results shown in [63] demonstrate a multiple initiative collaboration methodology, where the robot can wait to understand the human intentions and can drive the task once it understood. Figure 6 represents a diagram of the distinct layers that compose the cooperative procedure developed in [63]. It is easily deducted that the procedure represented in Figure 6 could be applied to most of the human-robot co-working systems. This exchanging of roles engages a very natural way of collaboration, allowing the users to only focus on their own part. The work presented in [63] results in an easy and intuitive physical interaction for an efficient Human-Robot Collaboration.

In [4], it is possible to clearly see a separated study about these two kinds of physical interaction: reactive compliance and proactive compliance. In reactive compliance, the robot feels the force exerted on its body and moves in a way to minimize the forces. Despite the apparent simplicity of this concept, there are some issues that could prevent an effective and expected robot movement, such as identifying the point where the human contact occurred and understand if the contact was accidental or deliberated [64]. Some studies, such as [65], help the robot in its haptics, making it more aware of the correct position where the contact force was exerted. A reactive compliance behavior can also be hard to obtain when the forces are exerted in a object that the robot is holding rather than directly in the robot’s body. Notwithstanding the work that tried to tackle this problem as, for example [66], this issue is still far from being efficiently solved. In a more evolved overview, predicting human’s intentions through the exerted forces applied to the robot body has gathered many robotic researchers. According to [4], the challenge of this search for a proactive compliance is related to the accuracy of the inferences made from the exerted forces. The authors of these mentioned investigation point out some important considerations regarding this particular topic: developing better prediction abilities in collaborative robots allows to decrease the physical and mental load on the human operator; nevertheless, the probability of unexpected robot movements, which compromises the success of the task, increases. For this reason, ref. [4] defends that a more clear-cut between autonomy and reactive compliance, i.e., a balanced solution, would be the best suitable option to efficiently use collaborative robots to ease human tasks.

## 3. Current Advances and Trends

Collaborative robots are being seen as the next big step in assisting human operators to complete cognitive tasks. This recurrent interest in developing machines capable of working in a similar way as humans has increased the number of research works and it has strengthened the scientific outcomes that recent HRC studies have developed. The main current trends that this topic encloses are usually related to the exchange (give/receive) of objects with the robot, the artificial intelligence that could be used for the machine to learn from humans and with the proper evaluation metrics to correctly analyse the performance of the collaborative robot. In this section, each one of these mentioned advances will be explored.

### 3.1. Object Handover

There are many concrete applications, where a robot has proven to be useful in helping humans by handing over objects, such as short-cycle repetitive tasks [67] or dynamic object handover tasks [68,69]. In the last decade, some studies described the process of object handover between humans with the purpose of transferring that same execution to robots [70,71]. However, that idealization of disassembling the handover process into different parts, giving separated instructions, has been discussed in a way to consider the handover as a single process [72]. There are some issues regarding the topic of object transfer between humans and robots.

The uncertainties about the geometry and physical properties of objects emerge as an adversity for the robot to understand how it should grab the object. To overcome this concern, object pose estimation [73], grasp adaptation strategies [61] and tactile feedback systems [74] were developed. Nevertheless, for optimal object grasping, the robot should detect the shape, orientation, and possibly the mass center of each object. This detection must ensure a correct grasp for both static objects (robot as a giver) and in human hand objects (robot as a receiver). From the point of view of robot-to-human handover, an evaluation of proper object poses is discussed in [75].

Apart from the object itself, the point where the handover procedure will happen must be estimated; to predict this object transfer point, several studies on this theme have already been carried out [76,77,78]. Nonetheless, the proposed approaches of those works have some limitations: some studies only consider the robot as a receiver, others just as a giver. To tackle this issue, as an example, when the robot plays the giver role, it should estimate, through the human body dimension, position, and orientation, the best spatial point to deliver the object. This estimation could be successfully achieved by the adaptation of the optimization works that were already developed regarding this concern. Since a natural object transfer will rarely occur statically, the trajectory planning for giving or receiving an object must be continuously evaluated and planned [79]. For the robot to receive a continuously moving object (in the human hand), the trajectory to reach that object must be re-planned accordingly.

To achieve a natural object transfer, the robot has to understand human intentions, by communication strategies apart from the physical contact. Human eye gaze cues are considered important to be detected and interpreted by the machine [80,81]. Human-Robot Collaboration future development aims to integrate robots in human teamwork, ensuring the continuity of a fluent workflow. This means that the robot should give and receive the objects in a similar way as humans do. The human-like motion will result in a more comfortable collaboration with robots [82,83], through the robot speed and reaction adaptability [84,85]. Moreover, non-verbal communication could be used through physical properties for a human intention understanding [86]. Some other studies have taken human safety into a big consideration, developing grasping selection and reaction systems that avoid the collision with the human hand [87,88,89].

**Figure 7:**
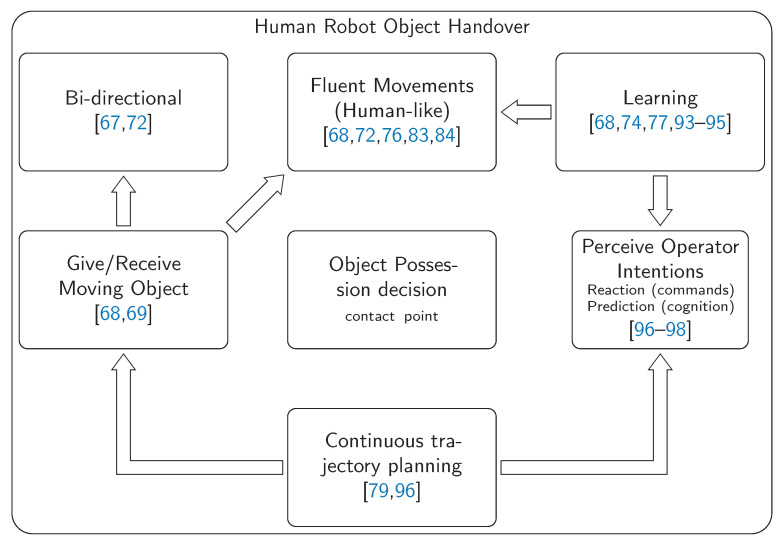
Human-robot object handover challenges [67,68,69,72,74,76,77,79,83,84,93,94,95,96,97,98,96].

Focusing on the ultimate moment of any object handover procedure, when both agents are in contact with the object, the coordination of the object weight transfer should be ensured. This stands as the critical instant where the giver should make sure that the object will not fall [90,91,92]. Furthermore, for a dynamic handover, the giver should also be prepared for a possible regret from the receiver to take the object possession. At this point there is no giver or receiver agents, since, when both of them are in contact with the object, the human operator could want to get the object for himself or let it to the machine, despite the initial object possession. All of these discussed topics regarding human-robot object handover tasks can be represented in Figure 7.

### 3.2. Robot Learning

It is a well-known fact that pre-programmed functions for a robot will narrow its capabilities. For environments where the purpose of the machine is to simply perform some repetitive tasks, this pre-programming strategy is more than suitable. However, when it comes to creating a collaborative robot, able to fluently execute dexterous tasks in coordination with a human operator, machine learning techniques development emerge as the best solution to achieve this goal. This kind of techniques can be implemented for proper recognition of all the scene that surrounds the robot (i.e., its human co-worker communication and its environment) and its respective classification. The correct classification of the surroundings allows the machine to easily predict what will probably happen in the near future, helping to anticipate the human motions, creating a more fluent task accomplishment. Moreover, the robot could also learn, through artificial intelligence methodologies, to move like a human, by transferring captions of human motion to the robot’s space motion.

By analysing sensory data from each human-robot collaborative system, it is possible to extract strong clues about the human co-worker. As an example, ref. [99] uses a laser range finder sensor to detect and track the human legs in order to recognize gait patterns. In [99], an adapted Hidden Markov Model (HMM) was developed to obtain an appropriate state estimation of human walk. Another example is presented in [100] where the HMM was used to estimate human affective state in real time by collecting data of heart rate, perspiration rate, and facial muscle contraction from several humans. For this type of classification, deep learning has proven to be the most recurrently used technique. The work present in [98] focuses on studying how deep representation learning should be used for human motion prediction and classification. In [101], a hand-eye coordination model for grasping was developed through deep learning and data collection. This study revealed the potential of this approach since novel objects, which were never considered during the training sessions, were successfully grasped by the robot. However, this type of procedure is widely known for requiring a huge amount of data to be considered effective. This concern appears as a shortcoming for using deep learning in other wider purposes. The work in [24] presents a deep learning framework for human motion capture data. This framework learns a generic representation from a extensive motion capture data and generalizes to new, unseen, motions. Once more, this type of approach has the shortcoming of requiring a large corpus of captured data in order to obtain acceptable results.

Besides the human motion classification, one of the most relevant research within this topic of interest relies on human anticipation [96]. Predicting human movement highly impacts the user experience of collaborating with robots. This anticipation will give much more fluency to the performed task [97]. The capability of predicting human motion will promote a proactive system with high coordinated actions. To this end, several studies were already developed where machine learning is deeply discussed as the key tool for the optimal collaborative robot task performance [8,93,94,95].

Apart from the perception and recognition of the environment, the robot must also plan its trajectory to achieve each proposed objective. For a collaborative robot, the action behavior should be synchronized with its human co-worker and fluent, similar to a human movement. This type of motion planning and control is hardly achieved through the classical analytical methodologies. For this reason, reinforcement learning has started to be implemented in HRC systems, aspiring to provide the tools for bringing the robots motion closer to human motion. Since reinforcement learning is based on knowledge acquisition by trial and error, at the beginning it is expected that the robot moves like a new born being, without knowing anything about the real world. By measuring several cost functions, the machine will learn the decisions that return the lowest possible cost. Even though the good strategy that this type of machine learning provides, a large amount of time is required, as well as patience and effort to achieve the moment where the robot starts to be useful for collaborating with humans in certain tasks. To avoid this long wait for the robot to behave nearly to what is intended, it is possible to provide few and limited data through which the robot will begin to learn by itself [4]. This combination of demonstration data with reinforcement learning pop up as one of the most efficient solutions to reach the human-like robot motion without requiring heavy amount of data neither huge amount of time. This combination is addressed as learning from demonstration.

In [102], a collaborative robot learns to lift an object through a previous demonstration by the user by controlling the robot’s hand via a haptic interface. The teleoperation of the robot’s hand provides dynamic information that need to be broken down to be understood by the robot. This analysis is performed using a probabilistic model based on Hidden Markov Model and Gaussian Mixture Regression. Sidiropoulos et al. [103] had also used Gaussian Mixture Models (GMM) and haptics to achieve a fluent robot-to-human object handover. In this work, the authors had demonstrated the human wrist motion of the object handover approaching phase. For this demonstrations, a dynamical system was used to capture the position and orientation of the human wrist, which was then mirrored to the robot motion. Another research development that uses both training datasets and execution behavior as sources for teaching the robot is presented in [104]. The robot used in the study could start acting autonomously based on a Markov Decision Process, where some reward functions would dictate the confidence on the task that the robot was about to perform.

One of the challenges related to human demonstration for robot learning is encoding the human motions into robot understandable parameters. In addition to this synthesis, it is also necessary to exclude the extra parameters (included in the complex human motion) that will not provide useful information for teaching the machine. There are a few methods to handle this concern such as the Dynamic Motion Primitives (DMP), which was used in [105]. The use of this technique enables the human motion representation and its extent into Cartesian space. This extension will ease the transfer of human movements to the robot’s joint space. In [94], the encoding of human demonstrations is achieved by a task-parametrized version of the Gaussian Mixture Model. These authors specifically propose a framework for a user to teach a robot collaborative skills from demonstrations.

Lastly, ref. [106] addresses this model-based and model-free combo learning as deep reinforcement learning, inferring that this type of approach is limited to simpler tasks, being ineffective in high-dimensional dexterous manipulation. Rajeswaran et al. [106] have effectively scaled up the usage of deep reinforcement learning and human demonstration to teach a complex manipulation task to a high-dimensional 24-dof robotic hand, such as nail hammering.

### 3.3. Metrics in HRC

Another current interest in Human-Robot Collaboration is how to measure the effectiveness, efficiency, fluency and adaptability of one these HRC systems. The definition of adequate metrics and guidelines to evaluate human-robot interaction tasks’ performance is, in [3], recognized as a truly important aspect to lead future developments.

Several research studies have focused on the definition for metrics for Human-Robot Interaction [107,108,109,110,111]. In [107], the metrics related to HRI are subdivided into several particular domains such as productivity, efficiency, reliability, safety, and co-activity. This metrics categorization is easily understandable through the natural complexity that every Human-Robot Collaboration procedure entails. It is difficult to assess such systems in a global and objective evaluation. In fact, many of the studied metrics in [107] cannot be generalized to an open mechanism for measuring the feature of its category. Hoffman et al. [110] discuss four specific metrics for evaluating human-robot fluency: the percentage of concurrent activity, the human’s idle time, the robot’s functional delay and the robot’s idle time. These metrics are general and agnostic to the specific content of the collaborative acts, relating only to periods of activity. Anyhow, the metrics were used to evaluate an objects operation task in a human-robot shared workspace. More specifically, regarding the human-robot object handover domain, the survey from [112] reports a comprehensive list of qualitative and quantitative metrics and identifies the major areas of improvement to reach performance comparable to human interaction. However, none of these metrics seems to cover completely every human criteria about the fluency and efficiency of a collaborative task performance. For example, the metrics presented in [110] do not fit every collaborative activities, since many simplifying assumptions were made such as the assumption that the time of the whole task is the same for the human and for the robot. Besides this, the authors of [110] declare that the presented metrics does not cover some aspects of physically coupled collaboration. Another interesting reported issue in [112] is that the large majority of human-robot object handover studies use only a single object class for the experiments: most of this objects were bottles or boxes. For a potential work with the purpose of generalizing the handover procedure to any possible object, a metric to separate different object in classes (through some descriptors), and to measure the success rate for each object class, is required.

The chosen metrics for human-robot object handover procedures should consider numerous factors, and several works have already created and used some measurement tools to analyse quantitatively and qualitatively the task of transferring objects between robots and humans. In [108], the evaluation of the object handover procedure is built upon a simple success/failure rate by measuring how many times the object was delivered within the total times of trial. This is an example of a basic quantitative metric, since the quality of the interaction was not evaluated. For the purpose of developing a fluent and human-like collaborative robot, qualitative and specific quantitative metrics are required, in order to interpret where and how can the system be improved and which methodologies should be refined or even replaced by new ones.

To tackle this concern, ref. [109] proposes a mixture between objective and subjective metrics to evaluate human-robot object handover interactions. The subjective metrics were formulated based on a score that each different participant gave after the experience of changing objects with the robot. Each participant had to evaluate four distinct aspects: how easy was to receive the object; how satisfied they are with the interaction; how comfortable was the interaction and how safe they felt during the interaction. Additionally, ref. [109] also takes in consideration some objective parameters, such as the effort per robot joint, the position and orientation of the human hand as a function of time, and many timing measurements related to the object handover practise.

Table 2 summarizes some works that use specific objective metrics, such as success rate [70,77,89,108,109,113,114,115], interaction force [72,90,91,116,117], timings [70,80,87,110,114,118,119,120,121], joint effort [109] and subjective metrics, such as fluency [70,80,87,110,114,122,123,124], satisfaction [109,113,115,123,125,126], comfort [91,109,115,121,124,126,127], usage of interaction [90,109,113,115,125,126], trust in the robot [87,110,125] and human-like motion [121] to evaluate their systems, providing a glance of the major trends within this field. The lack of generic metrics and benchmarks that can be applied to multiple Human-Robot Collaboration systems and even, ultimately, to human teamwork, holds an open path for future research and developments regarding this evaluation field.

The complete and reliable integration of human-robot collaborative systems into today’s world industry will only be achieved when it becomes possible to provide a common basis of comparison between systems, allowing for reproducible and repeatable evaluations. The aimed fair comparison demands an evaluation of many aspects, each one of them encompassing several parameters. Concretely, for a proper evaluation of a human-robot collaborative task, such as object handovers, the developed metrics should classify the robot motion fluency, the seamlessness with human motions, the coordination with the operator movements, the adaptive motion, the success in both giving and receiving an object, among others. These are the current research prospects, regarding evaluation metrics, among the HRC community. In this context, a suitable set of benchmarking test beds need to be developed for evaluating human-robot teams in a set of different scenarios.

## 4. Future Challenges

As it is possible to understand from the literature review discussed earlier, many methodologies and technologies were already created and applied within the Human-Robot Collaboration area of interest. Nonetheless, there are still several challenges to be tackled in this field, in order to achieve an intuitive, useful and effective use of collaborative robots for industrial tasks or even for general human-life improvement. Object handover among humans and robots constitutes one of the most important challenges to perform collaborative tasks in industrial environments, where the aimed high-level performance requires significant effort for designing suitable handover controllers in real-world scenarios. In these scenarios, the adaptation to the user’s needs and the user’s expectation in terms of robot behavior (i.e., robot acceptance) are decisive factors for an effective coexistence [91,128,129,130,131]. This points to the integration of cognitive abilities (e.g., interpretation, prediction, and learning) with perception and interaction abilities, which promises to create robots able to interact meaningfully with their human counterparts. The integration of cognitive abilities within a handover system emerges as another challenge to be considered in the near future research on Human-Robot Collaboration.

### 4.1. Open Research Questions in Object Handover

Several recent works have already developed partial solutions regarding the human-robot object handover procedure [132,133,134], some of which are inspired by human strategies [83,103,135,136]. However, none of them provides a generic solution for a human adaptive system, where the exchange of objects occurs fluently, similarly to what happens between two humans, for the robot to become a much more reliable and useful machine. For this purpose, several issues should be explored and developed in the future.

To pass an object to its human coworker, most of the collaborative robots have a fixed 3D spacial handover point, or pre-plan this handover point, in accordance to human body perception (size, position, orientation). This leads the human to adapt to the movements of the robot, which limits the aimed fluency. The robot should be capable of delivering an object in the hand of its coworker even if the coworker is not looking. This kind of handover occurs frequently between humans, especially in tasks that require an absolute concentration with any sight miss, such as, for example, performing surgeries. For this, the robot should execute a continuous perception of its surroundings and the human, in order to constantly re-plan the handover point until reaching the contact moment.

Furthermore, by the moment when both agents are simultaneously in contact with the object, the robot has to understand in real time who has possession of that object. For this purpose, payload changes and momentum sensibility must be taken into consideration to develop contact interfaces where the robot and the operator can communicate. The development of this issue will be useful for both situations where the robot plays the giver or the receiver role. When contact is mutual, there is not a giver and a receiver, since human intention can change at any instant. The machine should be prepared for contexts where the human may no longer want to receive that object that the robot is offering in that precise moment, or where the human reconsiders giving an object to the manipulator.

Object grasp strategies are also underdeveloped when compared to efficient human-human handover. Apart from the generic object shape gripper adaption, the robot should analyse which part of the object will the human need to grab in order to perform its task without the need for adjusting the hand for grabbing the tool. This requires a deepest understating of each object composition, sectioning the parts where the human will wants or not to receive that same tool.

### 4.2. Limitations and Opportunities for Cognitive Collaboration

Cognition is an integrated process that can encompass the entire robotic system and whose development depends on the desired level of interaction and the application domain. A collaborative robot can be endowed with diverse abilities to support greater autonomy and promote acceptance by the human partner. An important stage is the interpretation of the environment, including the human commands provided in verbal or non-verbal form, the arrangement of objects in a scene, and the recognition and tracking of active subjects based on 2D or 3D data. Purposeful manipulation requires the ability to generate task plans in response to high-level commands, to adapt its plans to the dynamics of the environment (including the user’s actions), to understand how manipulate different objects, to envision its own actions and to reason about goals. From a practical point of view, a cognitive robot must be endowed with adaptive anticipatory behaviour based on the current state, the past experience, and the prediction of future conditions. Therefore, learning and prediction will be critical elements for collaborative robot systems operating in the real world. The high variability of industrial settings makes it almost impossible to have accurate models of the environment, the objects in it, or the skills required to manipulate them in advance.

As mentioned previously, existing research includes context-aware object recognition [69], gesture recognition [17], human patterns recognition [99], human action prediction [98], learning manipulation skills from human demonstrations [103], and fine-tuning learned skills by exploring efficient forms of reinforcement learning [106]. Machine learning methods are increasingly used in robotic applications due to advances in their mathematical formalization. Deep learning and reinforcement learning are among the most promising methods applied in collaborative robotics, with their own levels of maturity and different challenges for real-world applications [137,138,139,140,141,142].

On the one hand, deep learning is characterised by large neural network models with multiple layers of hierarchical representation [143,144]. These models have achieved remarkable performance in computer vision for creating representation of the world through automatic extraction of perceptual descriptors [145]. However, there are several difficulties that need to be overcome before they may be applied in real-word scenarios. First, deep learning requires large amounts of annotated training data. Second, the training dataset must capture the entirety of possible situations to generalize well when facing novel examples [146]. Third, neural networks do not interpret cause-and-effect or why certain correlations or associations exist. Sünderhauf et al. [147] discuss several current challenges for deep learning that arise from the specific requirements of robotics applications and the difficulties to scaling the problem to real-world sensors, data, and computational resources. Reducing the data-dependency and understanding causality are among the highest priorities in machine learning.

On the other hand, reinforcement learning offers a framework for designing robots that learn and improve their behaviour through the interaction with the environment [148,149]. One of the challenges of applying reinforcement learning in robotics is to deal with large state-action spaces and long horizons. Equally challenging is the sample inefficiency of these algorithms since learning a policy may need an impractical number of interactions, reducing often its application to simulation. Another difficulty is to specify a reward function that exactly capture the problem at hands. Finally, and not least, there is a need for parameterized function approximators in order to generalize between similar situations and actions, mainly in the case of complex environments. The main challenges of reinforcement learning based robotics are discussed in the review paper by Kober et al. [150], including different solutions to address them. Reinforcement learning and imitation learning, combined with deep learning techniques, start offering novel computational tools for robotic skill acquisition and control problems, such as robotic manipulation [56,151,152,153] and robot grasping [101,154]. These methods are attracting much attention since the robot can automatically learn skills from the sensory inputs with minimal engineering.

From the current state-of-the-art, it is clear that applying cognitive functionalities in the domain of Human-Robot Collaboration is not yet a straightforward achievement. Additional efforts are still needed to demonstrate the benefits and added value they may bring to the context of specific application domains. The following is a summary of some open questions and promising developments that require future research.

#### 4.2.1. Representations and Understanding of the World

The idea of learning to represent the world before learning a task is gaining more attention [155]. The focus should be placed on developing methods that go behind current approaches in terms of what is learned and how it is learned. First, to learn how the world works, the robotic system is expected to develop concepts such as gravity, dimensions, and object persistence over time. Once learned, these models can be used by the robot to understand the world and then, to learn the actions that allow it to accomplish the desired task with few trials and few examples. There are other related themes that should deserve attention in HRC research, such as interactive learning as a basis for self-supervised learning [156,157] and causal models to support explanation and understanding of the world [158,159].

#### 4.2.2. Hierarchical Task Decomposition

Manipulation skills, such as grasping and handover, exhibit a hierarchical structure that helps break down them into multiple goal-oriented phases, such that the primary task is hierarchically divided in simpler and more tractable problems. Learning compositional and hierarchical task structures is a problem far from being solved [157]. Therefore, the deployment of robotic systems that can decompose the main task into primitive elements and learn to plan complex movement sequences would be a valuable contribution.

#### 4.2.3. Skill Reusability

Transfer learning [160], multi-task learning [161] and learning-to-learn [162,163] are closely related concepts associated with the idea of how learning a new task can be accelerated through previous knowledge of other similar tasks. In the future, learning techniques should assist in the development of collaborative robots that perform a wide variety of tasks without training individual skills. Skill reusability is an open problem that presents numerous opportunities for simplifying and improving robot programming of collaborative robots by generalizing knowledge to new tasks and situations.

## 5. Conclusions

Innumerable tasks can only be performed by humans (instead of robots) due to the high degree of complexity, dexterous coordination, perception, interpretation and decision capabilities they require. Even in an industrial environment, where the presence of automated robots is already a well established reality, several objectives can only be achieved through the collaborative work of human teams. In the past two decades, collaborative robots have been increasingly studied, developed and integrated in various sectors of the industry. These collaborative robots are not completely agnostic to the surroundings of their operational space, being capable of adapting their movements to possible human interventions during the task performance. This adaptation has several advantages such as safety promotion and human guiding acceptance.

This study was composed of two main parts. The first one reviewed the evolution of Human-Robot Interaction (HRI) and Human-Robot Collaboration (HRC) conceptualizations, enumerating various interfaces through which humans are able to interact with robots. Moreover, and considering that physical contact stands as the ultimate form of interaction, physicality was also analyzed. All the referenced works regarding these topics symbolize the extensive developments of human-robot interaction. Every interplay channel, such as gesture, human motion, voice, haptics, EMG/EEG, GUI, and the perception of physical contact between the robot and an object, or a human, should be considered for future advancements in Human-Robot Collaboration, since every one of these subjects contains a reliable base for communication, safety, and grasping.

The second part of this study focused on the current trends for Human-Robot Collaboration, discussing the possible future challenges that this open field still holds on. The reality of industrial use cases can only be effectively covered up by exploring the conception of an adaptable handover controller that accommodates a wide range of situations. This exploration requires addressing several issues that can be synthesized as follows. First, we followed the premise that the more similar to human-human transfer of objects, the better will be the human-robot objects handover process. Nonetheless, there are several open questions to ensure a completely fluent and general object handover process between robots and humans assuming the behavior of the “giver” or the “receiver”. Some unresolved challenges are related to the general possible hands pose, object orientation, object shape and to the object possession decision. Second, the integration of cognitive abilities (e.g., interpretation, prediction, and learning) with perception and interaction abilities promise to create robots able to interact meaningfully with their human counterparts. Despite the implementation of distinct learning methods (deep learning and reinforcement learning), using several models (HMM, GMM, DMP), in collaborative robots, additional efforts are still required to overcome some concerns to handle with the real world high variability of industrial settings: modeling and interpreting world’s reality, dividing complex tasks in simpler checkpoints and learning to learn. Third, to perform a systematic study of current approaches to tackle the challenges of object handovers and their current limitations, it is important to understand how to evaluate the different solutions and if there is a lack of appropriate metrics.

Bearing all this in mind, the goal of near future Human-Robot Collaborations is to aggregate both cognitive knowledge (interpretation, prediction, motion planning and learning) and physical knowledge (grasping, object release, human contact) concerning the robot, making it skillful and qualified to help humans in collaborative tasks. Hopefully, future technological evolution in this domain will assist human life in a fruitful and healthy way.

## Figures and Tables

**Table 1:** Definitions regarding Human-Robot Collaboration and Human-Robot Interaction concepts, from several distinct sources.

Source	Definitions
[9]	**interaction**: action on someone or something else.**collaboration**: working jointly with someone or something.
[10]	**collaboration**: working jointly with others or together especially in an intellectual endeavor.
[11]	interaction includes collaboration.**interaction**: action on someone else.**collaboration**: working with someone, aiming at reaching a common goal.
[12]	**collaboration**: robot feature to perform complex tasks with direct human interaction and coordination.**physical interaction**: few nested behaviors that the robot must ensure (collaboration, coexistence and safety).
[1]	**efficient Human-Robot Collaboration**: robot should be capable of perceiving several communications mechanisms similar to the ones related to human-human interaction.
[2]	**physical Human-Robot Collaboration**: the moment when human(s), robot(s) and the environment come to contact with each other and form a tightly coupled dynamical system to accomplish a task.
[13]	**collaborative robot**: able to understand its collaborator’s intentions and predict his actions, in order to adapt its behavior in accordance and provide assistance in a wide diversity of tasks.
[5]	**HRC**: requires a common goal that is sought by both robot and human working together.**HRI**: the interaction between the human and the robot does not necessarily entail a common goal, thus falling in the definition of coexistence.
[6]	**coexistence**: when the human operator and the robot are in the same environment but do not interact with each other.**synchronised**: when the human and the robot work in the same space but at alternated times.**cooperation**: when the human and robot work on separate tasks, but in the same space at the same time.**collaboration**: when the human operator and the robot work together on the same task.

**Table 2:** List of works addressing distinct metrics for Human-Robot Collaboration.

	Metrics Descriptor	References
Objective Metrics	Success rate	[70,77,89,108,109,113,114,115]
	Interaction force	[72,90,91,116,117]
	Timings (idle & total)	[70,80,87,110,114,118,119,120,121]
	Joint effort	[109]
Subjective Metrics	Fluency	[70,80,87,110,114,122,123,124]
	Satisfaction	[109,113,115,123,125,126]
	Comfort	[91,109,115,121,124,126,127]
	Usage of interface	[90,109,113,115,125,126]
	Trust in the robot	[87,110,125]
	Human-like motion	[121]

## Data Availability

Not applicable.
